# Analysis of PLA Composite Filaments Reinforced with Lignin and Polymerised-Lignin-Treated NFC

**DOI:** 10.3390/polym13132174

**Published:** 2021-06-30

**Authors:** Diana Gregor-Svetec, Mirjam Leskovšek, Blaž Leskovar, Urška Stanković Elesini, Urška Vrabič-Brodnjak

**Affiliations:** 1 Graphic Arts and Design, Department of Textiles, Faculty of Natural Sciences and Engineering, University of Ljubljana, Snežniška 5, 1000 Ljubljana, Slovenia; diana.gregor@ntf.uni-lj.si (D.G.-S.); mirjam.leskovsek@ntf.uni-lj.si (M.L.); urska.stankovic@ntf.uni-lj.si (U.S.E.); 2 Department of Materials and Metallurgy, Faculty of Natural Sciences and Engineering, University of Ljubljana, Aškerčeva 12, 1000 Ljubljana, Slovenia; blaz.leskovar@ntf.uni-lj.si

**Keywords:** polymer composites, nanofibrillated cellulose, lignin, polymerised lignin, agglomeration

## Abstract

Polylactic acid (PLA) is one of the most suitable materials for 3D printing. Blending with nanoparticles improves some of its properties, broadening its application possibilities. The article presents a study of composite PLA matrix filaments with added unmodified and lignin/polymerised lignin surface-modified nanofibrillated cellulose (NFC). The influence of untreated and surface-modified NFC on morphological, mechanical, technological, infrared spectroscopic, and dynamic mechanical properties was evaluated for different groups of samples. As determined by the stereo and scanning electron microscopy, the unmodified and surface-modified NFCs with lignin and polymerised lignin were present in the form of plate-shaped agglomerates. The addition of NFC slightly reduced the filaments’ tensile strength, stretchability, and ability to absorb energy, while in contrast, the initial modulus slightly improved. By adding NFC to the PLA matrix, the bending storage modulus (E’) decreased slightly at lower temperatures, especially in the PLA samples with 3 wt% and 5 wt% NFC. When NFC was modified with lignin and polymerised lignin, an increase in E’ was noticed, especially in the glassy state.

## 1. Introduction

Three-dimensional (3D) printing technology (also known as additive manufacturing (AM)) is constantly expanding, mainly due to the advantages in the rapid and cost-effective production of complex, tailored-shape products [1,2]. In order to meet the demands of an increasingly challenging market (automotive, aerospace, biomedical products, architectural and ornamental products, and other fields of application) [2], different materials have been used; e.g., polymers, metals, resins, ceramic, sand, wax, etc. The most commonly used polymers for 3D printing are poly(lactide) (PLA), acrylonitrile butadiene styrene (ABS), polyamide (PA), and polycarbonate (PC) [3,4,5]; however, in recent years, more sustainable and environmentally friendly materials have been sought and gradually introduced, such as collagen, alginate, chitosan, and cellulose and its derivatives (hemicellulose, lignin, etc.) [1] in terms of composites.

PLA belongs to the group of hydrophobic biodegradable aliphatic polyesters produced from renewable resources (corn, wheat, rice). Its advantages are a slow degradation rate, hydrophobicity and chemical inertness [6], high strength, and high modulus [7]. Besides its advantages, it also has some drawbacks; e.g., inherent brittleness—low toughness, low thermal stability—its hydrolysable backbone can be easily degraded by thermal processing and hydrolytic reactions, which results in a decrease of molecular mass and worsening of mechanical properties [8,9]. Reinforced with different types of nanofillers, properties of PLA nanocomposites could be improved; however, cellulosic nanomaterials, among which is also nanofibrillated cellulose (NFC), attract a lot of attention.

Biodegradable NFCs, obtained from plants or other microorganisms, have low density, high strength, and high specific surface area and crystallisation, and can be used as reinforcement in different materials and products (biomedical [10], smart textiles and packaging [11], sensors and ion sensors [12], pulp and paper, paints, films, coatings [3], etc.). As described by Shin et al., NFC has been used as a biomedical printing platform for 3D printing [10]. The advances in 3D printing from renewable materials were also presented by Markstedt. Their research presented the NFC and xylan dispersions as a printing platform that mimics wood biogenesis for the assembly of wood biopolymers into wood-like hierarchical composites, which could be used in many fields [11]. On the other hand, Kim et al. described in their study the use of NFC as a 3D-printable conductive ink developed and optimised with cellulose nanofibers by adding silver nanowires for sustainable and biocompatible sensor applications [12].

The demanding phase in the process of producing NFC from an aqueous suspension in a dried state (as is needed in thermoplastics processing) is drying. Žepič et al. proved that the drying process is intended to preserve the original morphological properties of the fibrillated cellulose and to avoid the changes in the polymorphic structure and deformation of fibrils [13]. As shown by some researchers, the drying techniques (e.g., spray-drying, freeze-drying, super-critical drying) have a significant influence on the size and morphological shape of dried particles [13,14]. Peng et al. chose spray-drying for drying NFC suspensions [14]. They proposed a two-particle formation mechanism during spray-drying in which fibrous particles and fibrous agglomerates were formed.

The composites based on the PLA matrix and NFC as the filler have been studied by different researchers. As described by Peng et al., nanocomposites, in comparison to conventional composites, have improved thermal, mechanical, barrier, and flammability properties even at smaller filler quantities (e.g., ≤5 wt%) [15]. However, it is important to emphasise that the improvement of nanocomposite properties depends on different factors such as the properties of the matrix and of the filler (especially percentage, shape, and size of particles, and their orientation in the matrix), the filler–matrix compatibility and interactions, and the dispersion of the filler in the matrix for fibre-reinforced systems. Wang et al. found that hydrophilic cellulose nanofibrils (CNF) with a polar surface and a hydrophobic non-polar PLA matrix do not form strong interfacial interactions [1]. However, this interaction can be improved with the presence of reactive functional groups on the nanoparticle surface, as described by Mokhthu et al., Murphy et al., and Collins et al. [16,17,18]. Thielemans et al. found that one of the surface treatment agents for nanocellulose fibres is lignin, a low-cost compatibilizer [19] with interesting properties such as antiaging, flame retardancy, antioxidant capability, and UV absorption. Lignin is composed of repeating phenyl propane units and aromatic hydroxyl groups, while its carboxylic acid groups are important for compatibility between CNF and PLA through hydrogen bonding and van der Waals interactions. When the surface of CNF is treated with lignin, the initial dispersion is improved, and the reaggregation of CNF in the PLA matrix is prevented, as presented by Wang et al. [20]. Wang et al. used lignin-containing cellulose nanofibrils (LCNFs, with an average length of 400–600 nm and width of 12–20 nm) for reinforced PLA films (obtained by solution casting) [3]. According to the results, they concluded that adding a relatively small amount of LCNFs to PLA can change its chain mobility within the glass-transition region, implying that some strong interfacial forces are established between PLA and LCNFs (lignin is connected to cellulose through hydrogen bonds and dipole–dipole interactions, while it generates van der Waals interactions with PLA). Therefore, the compatibility between CNF and PLA improves through lignin. There are several studies presenting PLA/CNF/lignin composites, and no study was found presenting the mentioned composites with the polymerized lignin.

In this research, composite filaments of PLA as the matrix and nanofibrillated cellulose (NFC) and surfaced-modified NFC by lignin and polymerised lignin were analysed. The aim of this research was to study composite PLA filaments reinforced with (a) NFC, (b) surface-modified NFC by electrostatic adsorption of lignin (NFC + L), and (c) surface-modified NFC by electrostatic adsorption of lignin with post-surface enzymatic lignin polymerisation (NFC + PL). This research was a part of the project *Cel.Cycle*, in which individual input materials (NFC, NFC + L, and NFC + PL), and the final PLA and composite filaments (PLA + NFC, PLA + (NFC + L), and PLA + (NFC + PL)) were prepared by our partner as described by Božič et al. [21]. Our part in the research was to analyse the morphological, technological, mechanical, infrared spectroscopic, thermal, and dynamic mechanical properties of some raw materials and final products.

## 2. Materials and Methods

### 2.1. Materials

For the purpose of this research, 10 biocomposite filaments were produced. All filaments were composed of the same matrix polymer, high-molecular-weight biopolymer grade poly(lactide) (PLA) (Ingeo 2003D, NatureWorks, supplied by Resinex, Croatia). The commercial nanofibrillated cellulose (NFC; University of Maine, USA) used as an organic filler included nanofibers with a diameter of around 50 nm and about several 100 μm in length, as in Božič et al. [21]. Kraft lignin obtained from the pulp production as a byproduct in the paper industry (Andritz AG, Graz, Austria) was used for the NFC surface modification [21]. The NFC surface modification was performed in two processes that were described in an earlier work by Božič et al. [21]. Surface modification with lignin (L) occurred via electrostatic interactions between NFC treated with CaCO_3_ (Merck KGaA, Gernsheim, Germany) and lignin solubilised at alkaline pH. Finally, the pH was adapted to 4.5. With that treatment, a cationic charge on the surface of NFC was built. Modified NFC was washed by centrifugation several times. In the second process after surface modification via electrostatic interactions, lignin polymerization with enzyme laccase (Merck KGaA, Gernsheim, Germany) was performed, followed by washing by centrifugation [21]. Both NFC and lignin are strongly hydrophilic. The hydrophylicity of NFC changed after the treatment with lignin, and the contact angle increased from 25° to 50°, as reported Božič et al. [21]. The surface charge of NFC was slightly negative in the whole pH area, as determined by potentiometric titration. The overall surface charge increased after treatment with lignin/polymerised lignin, and the presence of functional groups with positive charge also was seen [21]. After the modification with lignin (L) and polymerised lignin (PL), the obtained dispersions (NFC + L and NFC + PL) were dried with the freeze-drying (lyophilisation) technique. The dried coarse powdery structure of the NFC, NFC + L, and NFC + PL samples were further ground by a rotor mill Retsch (Retsch GmbH, Haan, Germany) at 4500 rpm for 1 h. The unmodified and modified NFCs were compounded with PLA in different percentages (1 wt%, 3 wt%, and 5 wt%) on a Labtech LTE L/D = 4:1 twin-screw extruder (Labtech, Samutprakarn, Thailand) with a screw speed of 800 rpm and a barrel temperature of 155–157 °C. From the obtained granulates, final samples of filaments were produced on a Noztek Pro Desktop Filament Extruder (Noztek, UK) at a barrel temperature of 200 °C [21]. The screw speed could be set up to 2.5 m per minute, but was adapted to avoid flow instability and severe distortions of the extrudate, as well as to place it in the lower tolerance limit of 1.75 mm filament diameter. Before processing, the drying of granules in an oven (Memmert, Schwabach, Germany) at 90 °C was performed, until moisture content of <0.02% was reached. The samples in our study were marked according to the percentages of unmodified (NFC) or modified NFC (NFC + L or NFC + PL) added to the PLA matrix: PLA + NFC, PLA + (NFC + L), and PLA + (NFC + PL).

### 2.2. Methods

#### 2.2.1. Morphological Properties

For a surface and structure morphological observation, a Leica S9i stereomicroscope (Leica Microsystems Ltd., Heerbrugg, Switzerland) and scanning electron microscope (SEM JSM 6060 LV, Jeol, Tokyo, Japan) were used. The images from the stereomicroscope were captured with the program Leica Application Suite. For a morphological observation of the inner structure, composite filaments were (a) ruptured by hand, and (b) cut in the transverse and longitudinal directions with an IsoMet low-speed saw (Buehler, Lake Bluff, IL, USA) with sintered cubic boron nitride (CBN) cutting blades. All prepared samples were fixed on a specimen stub and covered with an ultra-thin coating of gold (with high vacuum evaporation).

#### 2.2.2. Technological Properties

The linear density of filaments was determined by weighing the predetermined length of filaments using an analytical balance (Mettler AE2000, Toledo, OH, USA) according to the EN ISO 1889:2009 standard. The diameter of filaments was measured using a micrometre (Mitutoyo, Sakado, Japan) with a foot pressure of 20 kPa acting on a sample according to the ASTM D 3218 standard. Density was calculated as the ratio of mass per unit volume. The time of the sound-wave propagation travelling longitudinally through the filament was determined using a PPM-5R pulse propagation meter (H. Morgan, Norwood, MA, USA). The measurement was performed at a frequency of 160 Hz at a distance of 10 cm with a 1 cm measuring step. The velocity of sound waves travelling through the filament was calculated as the ratio of the distance and the time of pulse propagation.

#### 2.2.3. Infrared Spectroscopic Properties

The Fourier transform infrared spectra analysis (FTIR) of samples was conducted with an ATR-FTIR spectrometer (Perkin Elmer, Waltham, MA, USA). The scans were performed in an infrared region of 4000–450 cm^−1^, with an average of 64 scans.

#### 2.2.4. Mechanical Properties

The tensile properties of filaments were determined using an Instron 5567 dynamometer (Instron, Norwood, MA, USA) equipped with a 10 kN load cell, according to the ASTM D 2256 standard, which prescribes the procedure for testing filament yarns. A modified procedure was used, whereby the measurements were made at a 100 mm clamping length. The specimens were tested at a speed of 10 mm/min. Tensile stress, elongation at break, energy at break, and initial modulus were determined, as well as stress, elongation, and energy at maximum force. Bending stiffness was determined with the 2-point bending method, in which the force acting on a clamped specimen was set at a distance of 50 mm. Compression force was measured by compressing the specimen, which was 50 mm in length, to half its thickness at a speed of 10 mm/min using an Instron 5567 dynamometer equipped with a 10 kN load cell. Modulus was derived from the stress–strain curve as the ratio of stress difference to the corresponding strain difference. For each method, 10 test specimens were tested at standard atmospheric conditions (22 °C, 50% RH).

#### 2.2.5. Thermal Properties

##### Dynamic Mechanical Properties (DMA)

The dynamic mechanical analysis (DMA) was performed using a Q800 DMA analyser (TA Instruments, New Castle, DE, USA). The measurements were performed in a dual cantilever bending mode on filaments that were 35 mm in length, at 10 Hz of oscillation frequency, 10 nm of oscillation amplitude, and a temperature step (ramp) of 3 °C/min in a range of 40–100 °C. The glass transition temperature (Tg), elastic modulus (E’), and tangent delta (tanδ) were determined.

##### Differential Scanning Calorimetry (DSC)

DSC was performed using a Q200 DSC instrument (TA Instruments, New Castle, DE, USA) equipped with a liquid nitrogen cooling accessory. The samples were first heated from 0 °C to 200 °C at a rate of 10 °C/min, followed by an isothermal step for 1 min to eliminate previous thermal history, and then cooled to 0 °C at a rate of 10 °C/min. After the isothermal step for 1 min, the second heating was conducted between 0 °C and 200 °C at a heating rate of 10 °C/min. The measurements were performed in a nitrogen atmosphere at a flow rate of 50 mL/min using aluminium standard pans with lids. The sample mass was between 5 and 6 mg. The cold crystallization temperature (*T*_cc_), melting temperature (*T*_m_), enthalpy of cold crystallization (Δ*H*_cc_), and enthalpy of melting (Δ*H*_m_) were determined. The degree of crystallinity (*X*_cc_) was estimated according to the following equation:

(1) $$X_{\mathrm{cc}}\left(\%\right)=\frac{\Delta H_{\mathrm{cc}}}{\Delta H_{0}\times X_{\mathrm{PLA}}}\times 100\left(\%\right)$$

where Δ*H*_cc_ refers to the cold crystallization enthalpy of the PLA and composites; Δ*H*_0_ refers to the enthalpy value of 100% crystalline PLA, which is 93.6 J/g; and *X*_PLA_ refers to the weight ratio of PLA in the composites.

##### Statistical Analysis

The obtained data were evaluated for statistical significance using a Student’s *t*-test. All results are displayed as mean ± standard deviation (SD). The differences observed between the properties of filament samples were considered significant when *p* < 0.05. The Pearson correlation coefficient was determined among the technological, structural, and mechanical properties of filaments, and separately for groups of filaments—GR1: PLA + NFC; GR2: PLA + (NFC + L); GR3: PLA + (NFC + PL).

## 3. Results and Discussion

### 3.1. Morphological Properties

**Figure 1:**
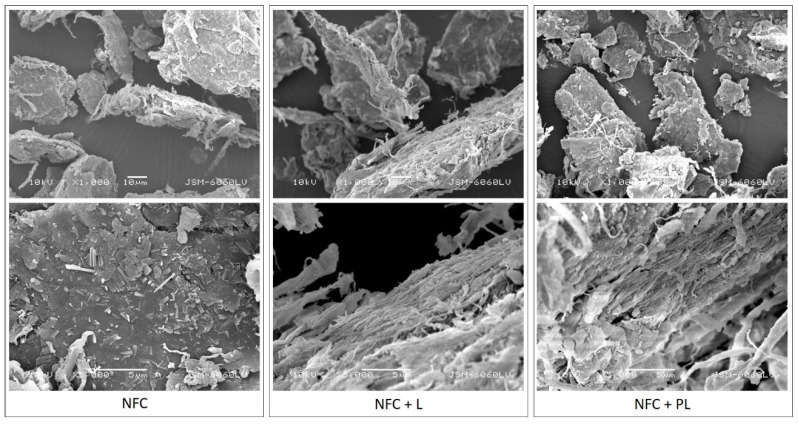
SEM images of NFC, NFC + L, and NFC + PL (magnification: 1000× (top) and 5000× (bottom)).

For a better understanding of the properties of composite filaments produced in our research, the used unmodified NFC, and NFC with lignin and with a polymerised lignin-modified surface (NFC + L and NFC + PL, respectively) were observed by SEM. In the images in Figure 1, it can be seen that the dried unmodified and modified NFC was in agglomerate form. The input material that entered the compounding phase had to be prepared in a dried state; thus, the dispersions of unmodified and modified NFC were previously dried by the freeze-drying (lyophilisation) technique. A common phenomenon that occurs during the freeze-drying of the nanofibrillated cellulose dispersion is agglomeration, which was proved by Peng et al. [22]. The agglomerates also appeared in our dried samples of unmodified and modified NFC. The dried powder was hence further ground to reduce the size of agglomerates.

Figure 1 shows that after the grinding, agglomerates were of plate-like irregular shapes in different sizes (up to 100 μm and more; note: size of agglomerates was roughly determined from images captured by SEM). The surface of agglomerates in the NFC sample was slightly smoother, with visible nanofibrils mostly protruding from the inside of agglomerates (especially in the areas ruptured due to grinding). In the NFC + L and NFC + PL samples, the surfaces of agglomerates were more fibrillated (more obvious in NFC + L) due to surface modification.

**Figure 2:**
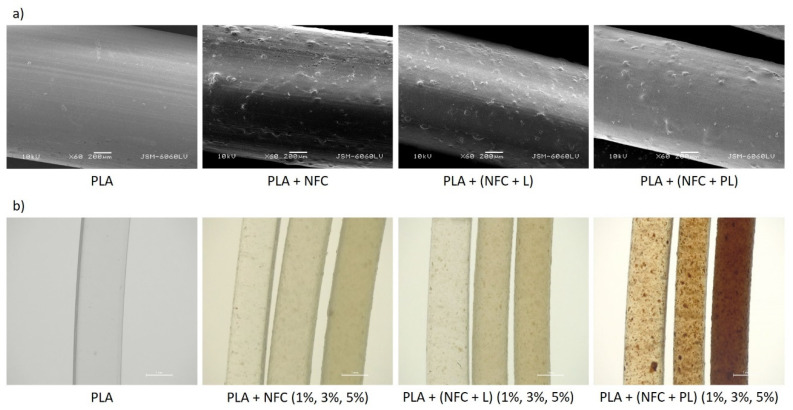
Surfaces of PLA, PLA + NFC, PLA + (NFC + L), and PLA + (NFC + PL) filaments captured by (**a**) SEM (magnification: 60×) and (**b**) stereomicroscope (magnification: 20×).

In all composite filaments, agglomerates were noticed on the surface (Figure 2a), resulting in an uneven surface structure. The agglomerates were unevenly dispersed and distributed through the filaments (Figure 2b). The composite filaments with unmodified and modified NFC were different in colour due to the colour of added fillers. PLA + NFC and PLA + (NFC + L) were slightly yellow, while the filaments made of PLA + (NFC + PL) were brown.

**Figure 3:**
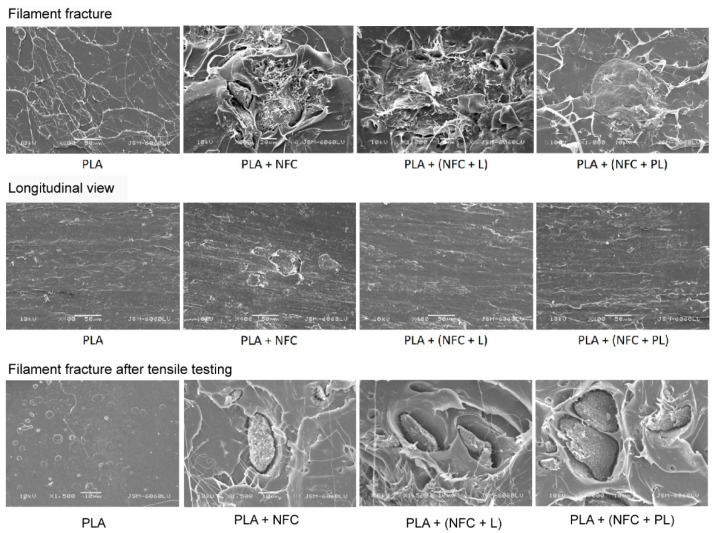
Filament fracture and longitudinal views of PLA and composite filaments PLA + NFC 3%, PLA + (NFC + L) 3%, and PLA + (NFC + PL) 3%; and fracture after tensile testing of PLA, PLA + NFC 5%, PLA + (NFC + L) 5%, and PLA + (NFC + PL) 5%.

The structure morphology of filaments was further studied in more detail by cutting and fracturing filaments in the longitudinal and transverse directions (Figure 3). Agglomerates were detected in the cross-sections of all fractured composite filaments (PLA + NFC, PLA + (NFC + L), and PLA + (NFC + PL)). Moreover, agglomerates were detected in the longitudinal view, but only in the PLA + NFC sample. It was assumed that in the composite filament with unmodified NFC (PLA + NFC), agglomerates were presumably oriented in both directions.

Although the modified NFC should have had better interfacial adhesion with PLA than the unmodified NFC, agglomerates were still not firmly connected with the PLA matrix. Slightly better adhesion and smaller air-filled gaps between the matrix and filler were noticed in the PLA + (NFC + L) filaments. The poorest adhesion between the matrix and agglomerates was noticed in the PLA + NFC sample, in which most of the observed agglomerates were completely surrounded with air-filled gaps that separated them from the PLA matrix, introducing weak points in filaments, which also confirmed Hubbe et al. [23].

In Figure 3, the fracture surface of composite filaments containing 5% NFC after tensile testing is shown. A smooth fracture with little roughness is seen in the PLA, which is a typical fracture characteristic of brittle materials. A short- and long-wire drawing appearance is present in composite filaments, which is an indication of toughening materials. Similar behaviour of PLA/NFC composites was reported by Xu et al. and Likittanaprasong et al. [24,25] Composite filaments show that some NFC particles were pulled out. Poor interface interactions were also noticed. Additionally, many holes with smooth edges were present in the PLA matrix, probably created by the debonding of particles.

### 3.2. Technological Properties

A common feature of composite filaments is a relatively large variation in the diameter and linear density (mass per unit length) (Table 1). The coefficient of variation in our case was mostly up to 10% and higher in four samples. In composite filaments with unmodified NFC (PLA + NFC), both properties exhibited approximately the same value or slightly higher compared to the reference sample (PLA). With the addition of modified NFC to the PLA matrix, both the linear density and diameter of filaments decreased. The lowest values were obtained in composite filaments when NFC + PL was added to the PLA matrix (PLA + (NFC + PL)). A decline in linear density and diameter was connected to the conditions at the extrusion process that were influenced by the change in the melt-flow properties caused by the added filler; i.e., modified NFC. The stable extrusion process was performed by lowering the diameter of the extruded filament.

The addition of unmodified and modified NFC resulted in higher density of filaments. The composite filaments with the addition of the lowest percentage of modified NFC (1%) reached the highest density (Table 2), which then declined at a higher percentage of added modified NFC; i.e., 3% and 5%.

Compared to the pure PLA filament, the sound-wave velocity (Table 2) was higher in all composite filaments, which suggests that they had a slightly higher order and increased orientation of structural elements in the longitudinal direction. The differences among filaments were low, with the highest value being reached by the filaments made with the addition of NFC modified with PL (PLA + (NFC + PL)). While the sound-wave velocity exhibited a slight decline when increasing the percentage of NFC in composites with added unmodified NFC, the addition of modified NFC showed the opposite trend. These results coincided with the results of the SEM analysis and could be explained by a better interfacial adhesion of modified NFC with the PLA matrix, longitudinal orientation of agglomerates, and voids surrounding them.

The probability, or *p*-value, determined for the technological and structural properties of the filaments indicated that significant differences were obtained when a different percentage of modified NFC was added to the PLA matrix in most cases. The addition of the highest percentage of modified NFC (5%) resulted in a statistically confirmed difference in linear density and thickness (*p* < 0.05). Only for the filaments with NFC modified with PL (PLA + (NFC + PL)), the addition in all three percentages led to a *p*-value below 0.05, whereas for other composite filaments, the *p*-value was higher. In the case of the results for sound-wave velocity, a low *p*-value (<0.05) confirmed that the addition of unmodified and modified NFC (NFC, NFC + L, and NFC + PL), regardless of the percentage added, had a significant influence on its value. An opposite result was obtained for the density of filaments, where in all composite filaments, higher *p*-values were obtained, meaning that the influence of added NFC on density was not statistically confirmed.

When comparing the technological and structural properties between the composites containing unmodified and modified NFC, the differences in linear density and diameter were statistically confirmed. On the other hand, regarding density and sound-wave velocity, we could not confirm that the modification of NFC led to differences that could be statistically proven.

### 3.3. Infrared Spectroscopic Properties (ATR-FTIR)

**Figure 4:**
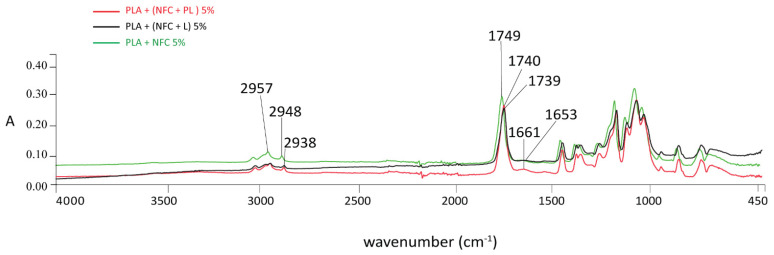
FTIR spectra of PLA + NFC 5%, PLA + (NFC + L) 5%, and PLA + (NFC + PL) 5% at wavenumbers of 4000–450 cm^−1^.

**Figure 5:**
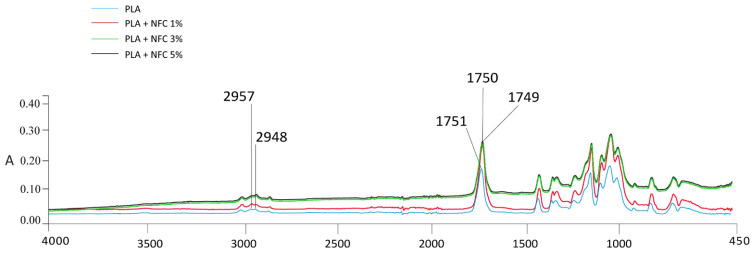
FTIR spectra of GR1 samples at wavenumbers of 4000–450 cm^−1^.

**Figure 6:**
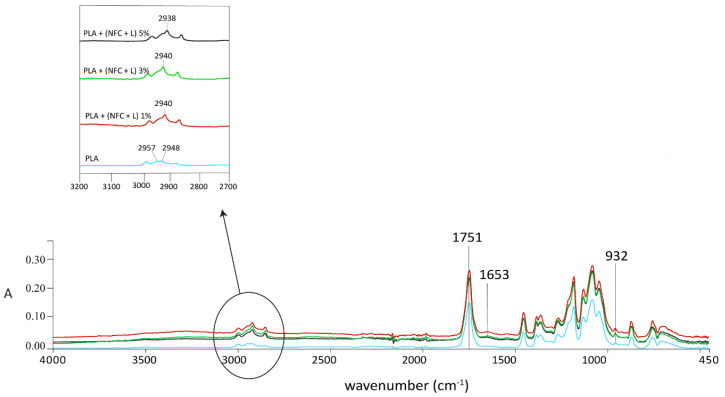
FTIR spectra of GR2 samples at wavenumbers of 4000–450 cm^−1^.

**Figure 7:**
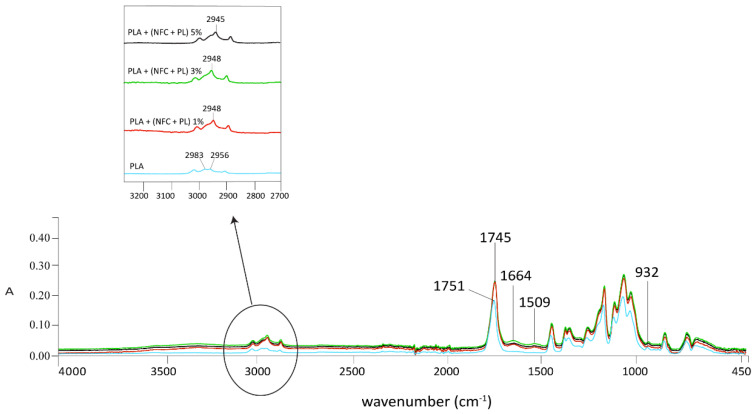
FTIR spectra of GR3 samples at wavenumbers of 4000–450 cm^−1^.

The infrared spectroscopic properties (FTIR spectra in Figure 4, Figure 5, Figure 6 and Figure 7) of the analysed samples in each group show the differences in absorption intensities. The analysis confirmed that the chemical structures of individual fillers influenced composite properties. At 1653 cm^−1^, the samples with lignin and polymerised lignin showed stronger and higher peaks, compared to the samples with only PLA and NFC (5%).

In the region of 2900–2980 cm^−1^, the sample with only PLA and NFC (5%) (Figure 4) was found to have two peaks; i.e., at 2957 and 2948 cm^−1^. These confirmed the presence of NFC and its C-H stretch. A strong peak at 2938 cm^−1^ was observed in the samples containing lignin and polymerised lignin.

In the region of 4000–450 cm^−1^, the intensity of the peaks of the sample with 100% PLA content was the lowest, while the addition of NFC caused an increased intensity of the peaks, as shown in Figure 5. The PLA/NFC composites had the same peaks as 100% PLA, except for the stronger peaks in the region of 3000–2940 cm^−1^. The C=O stretching vibration peak at 1751 cm^−1^ for PLA shifted by 2 cm^−1^ for the PLA/NFC composites. This could be associated with a weak molecular interaction between PLA and NFC, and this was not sufficient for strong interface interactions, which was also previously confirmed by Wang et al. [3].

The differences in absorption intensities were found in the GR2 sample group, as shown in Figure 6. Comparing the spectra of 100% PLA and samples with NFC and lignin, the intensity of the –C=O stretching peak at 1751 cm^−1^ for PLA shifted towards the low wave number by 12 cm^−1^ for the PLA + NFC + L 5% sample (1739 cm^−1^), which also was confirmed by Xiao et al. [26]. The intensity of the C=O peak increased slightly with lignin content, confirming the interaction between NFC, lignin, and PLA. This also suggests that lignin improved the compatibility between NFC and PLA [3]. The absorption peaks in the range of 1600–1500 cm^−1^ were related to the vibrations of the aromatic rings present in lignin, such as the weak absorption band in the range of 1585–1580 cm^−1^, which was associated with aromatic rings conjugated with an α carbonyl group, which was predicted by Inkinen et al. in previous research [27].

The addition of NFC + PL into the PLA matrix of the filament increased the intensity of peaks, as shown in Figure 7. The lowest intensity was found in the sample with 100% PLA. After the surface polymerisation of lignin, the peaks at 1509/1506 and 1661 cm^−1^, which belonged to the valence vibration of the aromatic ring, were more pronounced. However, the peak at 932 cm^−1^, which belonged to the C−OH oscillation, slightly decreased, indicating a possible connection of lignin via the ether bond. In all the analysed samples, both the intensity and appearance of peaks typical of NFC, L, or PL were more pronounced if the amounts of NFC, NFC + L, and NFC + PL were greater (5%).

### 3.4. Mechanical Properties

The data obtained from measuring tensile stress–strain properties of filaments were an important indication of the mechanical behaviour of the composite materials. The addition of NFC to the PLA matrix resulted in a change in tensile strength, initial modulus, elongation, and energy at break. The tensile strength of composite filaments was slightly lower (by up to 10%) compared to the tensile strength of the PLA filament, as shown in Table 3. With an increased NFC content in filaments, a decreasing trend was observed. The composite filaments with 5% of NFC added achieved, by up to 15%, lower tensile strength than the PLA filament, the lowest being for sample PLA + (NFC + PL).

Composite filaments were, in comparison to the PLA filament, less extensible; elongation at break, which reached 8% in the PLA filament, was lower in the case of composite filaments, and reached values of 4.2–5.9%. A similar influence of adding unmodified and modified NFC on the deterioration of the filament mechanical properties was seen in energy at break (Table 4). The work needed to rupture was lower for all composite filaments, regardless of the percentage of NFC added, meaning that the ability to stretch while absorbing the force was lower in composite filaments. In contrast to tensile strength, the initial modulus of composite filaments was higher compared to the PLA filament (3–13%), with the exception of two samples, in which a 4% lower value was determined. With a higher content of NFC in composite filaments, a trend of the initial modulus to decrease was observed, except in the case of the PLA + (NFC + PL) sample. The tensile properties of the filaments coincided with the results of the SEM analysis. The presence of agglomerates, their size, and their uneven distribution in the PLA matrix represented weak points in the filament, thus lowering tensile strength, stretchability, and tensile energy absorption ability. The composite filaments containing L-modified NFC (PLA + (NFC + L)) showed better interfacial adhesion between the modified NFC and PLA matrix than the other two groups of composite filaments, explaining their smaller deterioration of properties; i.e., tensile strength.

**Figure 8:**
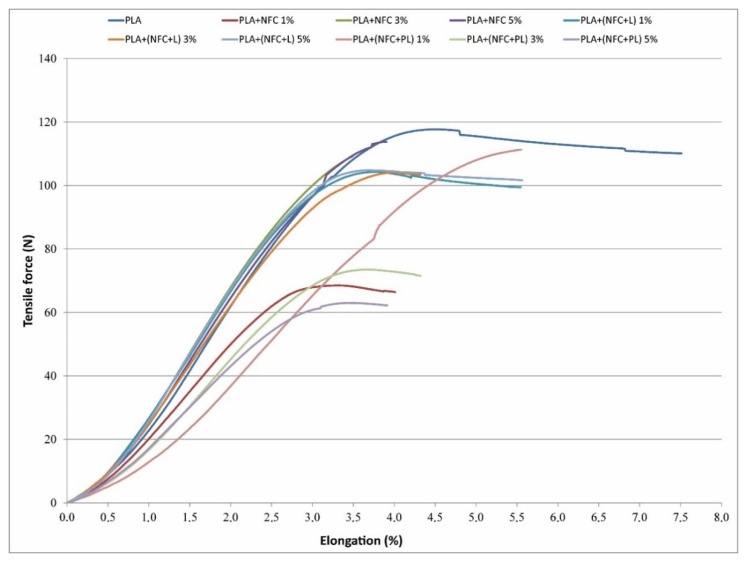
Tensile force vs. elongation curves of filaments.

As seen in Figure 8, not all filaments broke immediately after reaching the highest tensile force. They deformed under a slightly lower force until the breakage occurred. The difference between the maximum force and force at break, as well as stress and stress at break, was small; i.e., between 1% and 10%. A higher difference was in energy and elongation at maximum force, and in break; i.e., up to 50%, the highest being for the PLA filament, followed by the filaments with 1% NFC added. The addition of NFC decreased the stretchability of filaments, and their ability to absorb energy and deform without fracturing, in addition to lowering the maximum tensile force and tensile force at break. NFC as a hard filler dispersed in the polymer matrix acted as an impurity, causing discontinuity in the stress transfer across the filler—polymer interface.

Similar to tensile force, compression force was lower in the case of composite filaments, mostly by up to 10% (Table 5). The value decreased with a higher content of NFC added to the PLA polymer matrix. Furthermore, it was relatively substantially decreased for composite filaments with added NFC + PL, in which compression force was lower by up to 30%. Bulk modulus determined at the highest compression was shown by up to 10% lower values for composite filaments. In this case, we can assume that the addition of unmodified and modified NFC altered the compressibility of filaments due to the presence of agglomerates in different sizes and air-filled gaps between the matrix and the filler.

The force required to bend the filament, as well as bending stiffness, was lower in most composite filaments compared to the PLA filament, except in the case of the PLA + NFC and PLA + (NFC + L) samples, for which bending stiffness was higher (Table 6). The differences were rather small. Greater differences were obtained for the filaments produced with the addition of NFC + PL, where bending force was lower by almost 50% and bending stiffness by up to 35%. On the other hand, with the addition of modified NFC, the bending modulus reached higher values than the bending modulus of the PLA filament.

The correlation between the technical and mechanical properties of filaments was relatively low, with linear density and thickness showing correlation only with compression force (r = 0.92), bending force (r = 0.82), and stiffness (r = 0.69). Density did not show any correlation with the mechanical properties of filaments, whereas sound-wave velocity showed moderate negative correlation with the tensile properties in break (stress (r = −0.6), energy (r = −0.67), force (r = −0.75)), compression force (−0.72), and bending stiffness (r = −0.68). The correlation between different properties, determined separately for all three groups of composite filaments (GR1: PLA + NFC; GR2: PLA + (NFC + L); GR3: PLA + (NFC + PL)), was higher. Both linear density and thickness showed high correlation with tensile properties for all three groups; the Pearson correlation coefficient was between 0.6 and 0.95. For the group of composite filaments in which only unmodified NFC was added (GR1: PLA + NFC), the correlation was negative, which meant that tensile properties deteriorated with increased linear density and thickness of filaments. For the other two groups of composite filaments, containing modified NFC (NFC + L, NFC + PL), the correlation was positive. For the group of composite filaments with PL-modified NFC (GR3: PLA + (NFC + PL)), compression force and modulus, bending force, and stiffness showed very high positive correlation with the linear density and thickness of filaments (r = 0.92 to 1). The correlation between the density of filaments and tensile properties was seen only in the group of composite filaments with L-modified NFC (GR2: PLA + (NFC + L)), and was negative. In addition, a negative correlation was obtained with the compression and bending properties for the group of composite filaments containing only unmodified NFC. The sound-wave velocity showed good correlation with almost all mechanical properties for the group of composite filaments with PL-modified NFC (GR3: PLA + (NFC + PL)), the Pearson correlation coefficient being around 0.9. For the group of composite filaments with L-modified NFC (GR2: PLA + (NFC + L)), the sound-wave velocity showed correlation only with tensile properties. Moreover, in these cases, the correlation was negative, meaning that a higher-sound wave velocity resulted in worse mechanical properties.

**Figure 9:**
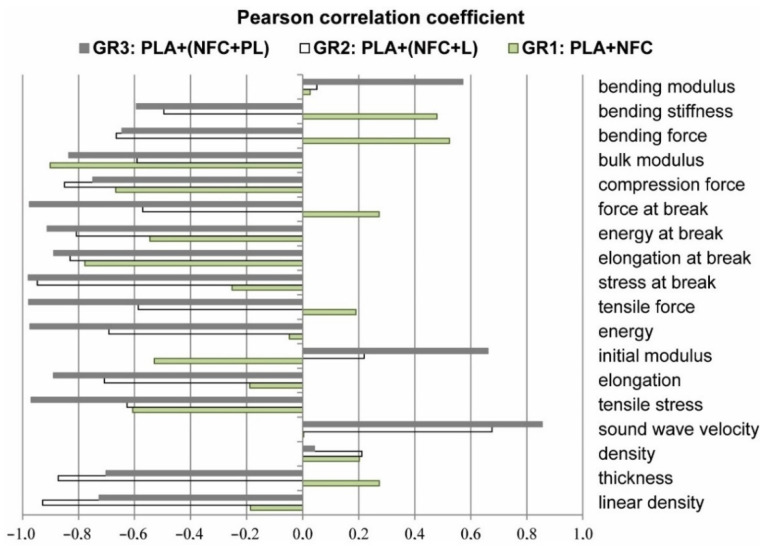
Pearson correlation coefficient between percentage of NCF and technical, structural, and mechanical properties of filaments—GR1: PLA + NCF; GR2: PLA + (NCF + L); GR3: PLA + (NCF + PL).

There was almost no correlation between the quantity of unmodified and modified NFC added to the PLA matrix and the technical, structural, and mechanical properties of composite filaments. The Pearson correlation coefficient, in most cases, was lower than 0.5; only for elongation and energy at break was it slightly higher (r = −0.76 and −0.62). If we look at the correlation inside the filament groups, the result was relatively different (Figure 9). The group of filaments with added NFC showed correlation only with compression force and modulus.

By adding modified NFC (NFC + L) into the polymer matrix, the correlation between the added percentage of NFC showed a negative correlation with the technical properties, tensile properties determined at break, compression, and breaking force of composite filaments (GR2: PLA + (NFC + L)). By increasing the percentage of NFC in filaments, the mechanical properties deteriorated. The highest correlation coefficients were determined with the group of composite filaments in which the samples contained modified NFC with PL (GR3: PLA + (NFC + PL)). The correlation there was obtained with almost all properties of filaments, with most being negative. A higher percentage of modified NFC led to filaments with lower breaking tenacity, stretchability, toughness, and resistance to compression and bending.

### 3.5. Thermal Properties

#### 3.5.1. Dynamic Mechanical Properties (DMA)

**Figure 10:**
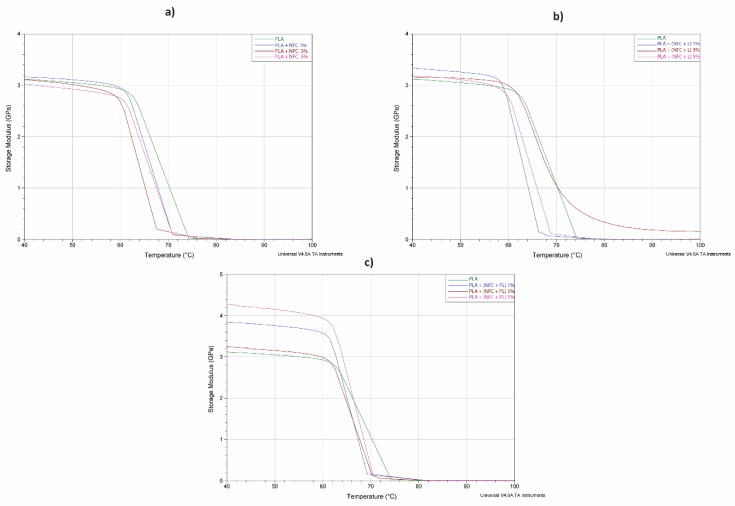
Bending storage modulus (E’) vs. temperature at 10 Hz of oscillation of filaments in groups (**a**) GR1, (**b**) GR2, and (**c**) GR3.

In Figure 10, the curves of the bending storage modulus (E’) vs. temperature are shown for all samples, while the values of the bending storage modulus (E’) at 40 °C and glass transition temperatures (Tg) are presented in Table 7.

As can be seen in Figure 10, the curves of the bending storage modulus E’ of all samples were similar in shape and typical of amorphous polymers. A rapid drop of E’ with increased temperature indicated in all samples a movement of macromolecules that tended to gain their equilibrium conformation state in a very narrow relaxation temperature interval.

In the glassy state (at 40 °C), by adding NFC to the PLA matrix, the bending storage modulus (E’) slightly decreased in two samples (PLA + NFC 3% and PLA + NFC 5%) and only slightly increased in the PLA + NFC 1% sample. When NFC + L and NFC + PL were added to the PLA matrix, a noticeable increase in the modulus was noticed for all samples (at 40 °C in Table 7). This increment could be attributed to a slightly better order and higher orientation of molecular segments with greater interactions among the main molecular chains of PLA, which also was proven by the sound-wave velocity measurements (Table 2) in the PLA + (NFC + L) and PLA + (NFC + PL) samples. Mahmood et al. [28] even suggested that the increment of elasticity could be explained by the molecular structure of polymerised lignin, which is a complex amorphous 3D cross-linked structure with no linear arrangement of repeating monomer units like other natural polymers; e.g., cellulose. During the process of lignification (lignin polymerisation), lignin bonds with the nanofibrils of the cellulose network, and raises their stiffness and the stiffness of composites. As stated, the elastic modulus of such cellulose nanofibrils is theoretically even higher than steel and comparable with Kevlar, ranging close to 137 GPa as described by Mahmood et al. [28]. The results showed that stiffness increased even more when NFC modified with polymerised lignin was added to PLA. Namely, at 1% addition of NFC to PLA, the modulus of elasticity increased by 21.5% (from 3.165 GPa for PLA + NFC to 3.847 GPa for PLA + (NFC + PL)); at 2% addition of NFC, by 4.3% (from 3.108 GPa for PLA + NFC to 3.241 GPa for PLA + (NFC + PL)), and at 5% addition of NFC, the elastic modulus increased by 41.7% (from 3.017 GPa for PLA + NFC to 4.276 GPa for PLA + (NFC + PL)). A further explanation for the improvement in stiffness also was given by Wang et al. [18] who suggested that lignin-containing cellulose nanofibrils (LCNFs) also acted as nucleating agents, resulting in an increased degree of crystallinity of PLA with LCNFs (the addition of lignin on the CNF surface could cause PLA chains to fold onto the LCNF surface, thus improving the interactions and compatibility between CNFs and PLA; lignin may act as a compatibilizer). From this, we can conclude that polymerised lignin also showed the strengthening effect in our research.

As seen in Figure 10, in the temperature region of about 60–75 °C, a rapid drop in the curves’ E’ of all samples was observed as a consequence of the segmental movement of the molecular structure due to the applied heat, while Tg was indicated as a peak of the tangent delta curves of samples. The pure PLA filament sample had the highest Tg among all samples (Table 7). When adding untreated NFC to PLA (PLA + NFC), Tg decreased. Wang et al. suggested that CNFs and PLA do not form strong interfacial interactions; therefore, it can be assumed that the added NFC formed free volume among molecular chains, contributing to a lower level of energy required to gain the movements of molecular chains in which the conformations of molecules reached their relaxation form state [20]. When NFC was treated with lignin and polymerised lignin, Tg was even lower than in the case of PLA and PLA + NFC. According to Wang et al. [3,20], chain mobility within the glass-transition region can change even with a small amount of LCNFs added to PLA. They noted that this indicates that strong interfacial forces are presented between LCNFs and PLA. However, some trends in our results should be mentioned as well. The Tg of individual samples (PLA + (NFC + L) and PLA + (NFC + PL)) negligibly increased with the increased percentage of NFC treated with lignin or polymerized lignin. This might again be a consequence of the rigid cross-linked 3D structure of the mixture of NFC and L/PL, which hinders the segmental oscillation and the occurrence of slippages of structural elements due to the applied heat. The conformational relaxation of the molecular structure thus occurs with a temperature delay, but as said earlier, this is limited only to some samples.

**Figure 11:**
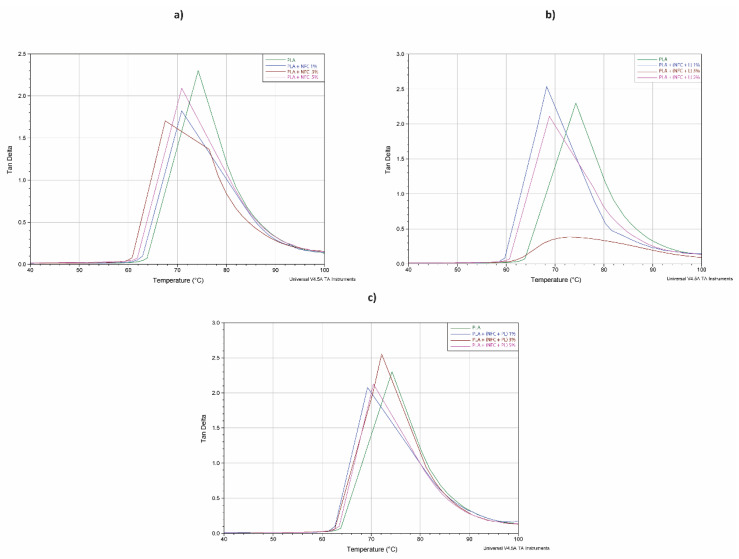
Loss factor (tan δ) vs. temperature at 10 Hz of oscillation of samples in groups (**a**) GR1, (**b**) GR2, and (**c**) GR3.

The curves of the loss factor (tan δ) vs. temperature of all samples are presented in Figure 11.

The values of tan δ peaks (Table 7) decreased with the addition of NFC, lignin, and polymerised lignin in almost all samples (compared to PLA sample) regarding their concentration, since the established interfacial forces mentioned before can restrict the movement of chains in amorphous regions.

The lowering of the damping could also be the result of the good filler dispersion in PLA matrix, which additionally restricted the segmental mobility of PLA chains, resulting in more elastic response of the filaments. A more detailed insight into the tanδ values of the composite samples also revealed that the higher peak intensity of samples PLA + (NFC + PL) was noticed. This could be the consequence of the increased occurrence of the reaction between NFC treated with polymerised lignin and PLA matrix that limited the crystallization of the matrix [29], which was also revealed in the DSC results given in the next subsection.

#### 3.5.2. Differential Scanning Calorimetry (DSC)

**Figure 12:**
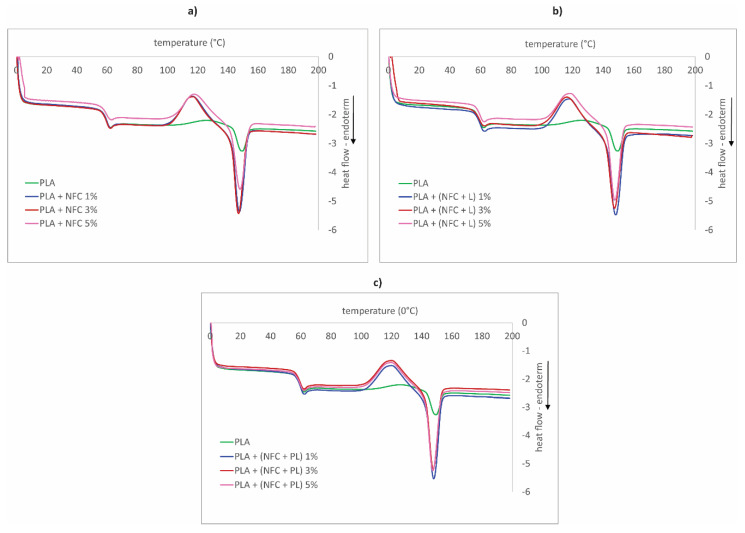
DSC second heating curves of samples in groups (**a**) GR1, (**b**) GR2, and (**c**) GR3.

The results of the second heating cycle of DSC measurements are presented in Table 8 and Table 9 and Figure 12.

As shown in Figure 7, the DSC curves indicated the three temperature transitions, which were typical for PLA polymer: the glass transition, the cold crystallization peak, and the melting peak. The area of the glass transition was located approximately at 60 °C (Figure 12), which was not in correlation with the glass-transition temperature indicated by the DMA analysis (Section 3.5). Since the DMA method was a more sensitive and suitable method for determining the glass-transition temperature, its results were more relevant. However, as in the case of the DMA analysis, it could be confirmed that NFC, lignin, and polymerized lignin (nor their content) did not significantly affect the glass-transition temperature of the filaments.

If not on the glass-transition relaxation, fillers had a significant effect on the cold crystallization region and caused the shift of the cold crystallization peak (*T*_cc_) to the lower temperature (approx. 126 °C in the case of PLA; Table 8) and sharpened it at the same time. The largest shift could be observed in the case of added NFC, and the smallest in the case of added NFC + PL. These results indicated the increased rate of PLA crystallization. A similar trend also was observed by Murphy and Collins [17] and Wang et al. [30], who reported that a faster crystallization rate of PLA occurred due to the nuclear role of NFC and lignin, which coincided with the results of the storage modulus and sound speed measurements of our research. The highest rate of crystallization of the PLA + (NFC + PL) filament also coincided with the increased damping (raised tanδ). Additionally, the content of fillers did not have any significant effect on the cold crystallization peak, meaning that it did not affect the increase or decrease in the mobility of PLA chains.

The cold crystallization region was followed by a melting area with one melting peak (in the case of PLA, the melting temperature is 149.3 °C; Table 7), in which a similar trend, as seen in the cold crystallization region, occurred: the melting peak became more pronounced when fillers were added. At the same time, the melting temperature practically did not change (only a minor decrease was observed) with the raised content of each filler. The same conclusion also was found by Perić et al. [31]. In contrast, the enthalpy of melting was greatly increased by the fillers, which again proved their role as a crystal nuclei that increased the rate of crystallinity of the filaments. The degree of crystallinity increased significantly, from 6% to over 20% with the addition of NFC. PLA has low crystallization potential, and addition of NFC enabled formation of crystal nuclei near the NFC, resulting in enhanced nucleation and crystallization ability, while the increase in the filler content did not have any influence on the crystallization ability.

## 4. Conclusions

Cascading use of biomass and use of byproducts helps to close materials loops, results in new advanced materials, and opens new opportunities. PLA is renewable, fully biodegradable, and the most usable bio-based polymer for 3D printing. To overcome some of its drawbacks, PLA can be blended with fillers, such as nanoparticles. In most cases, the physical properties of PLA composites are enhanced by NFC incorporation; however, no improvement and even a decrease in the physical properties of PLA composites have also been reported [31]. In our research, composite PLA filaments with added unmodified and lignin/polymerised lignin surface-modified NFC were analysed. It was shown that the addition of NFC decreased the stretchability, toughness, breaking tenacity, bending stiffness, and resistance to compression, and slightly improved the initial modulus of filaments. Although NFC slightly toughened the PLA composite, poor interface interactions limited this effect. The analysis revealed that mechanical properties were related to surface modification of NFC and its content in the PLA matrix. By increasing the content of NFC to 5 wt%, the mechanical properties deteriorated the most. With the addition of lignin/polymerised lignin surface-modified NFC, the deterioration of mechanical properties was minor, and an increase in the storage modulus was detected.

Although NFC slightly toughened the PLA composite, poor interface interactions limited the efficient toughness improvement. The presence of agglomerates in the form of plate-like irregular shapes, unevenly dispersed in the filaments, caused weak points in the structure of filaments, leading to deterioration of their mechanical properties. In addition, pure interfacial adhesion between NFC and the PLA matrix resulted in inferior mechanical properties of composite filaments. SEM images and FTIR analysis confirmed the interaction between lignin surface-modified NFC and the PLA matrix. This phenomenon was additionally confirmed by DMA analysis, in which the increase in elasticity was highly noticeable in the case of added modified NFC. No significant differences in thermal stability were detectable among filaments with unmodified and modified NFC but when compared with the neat PLA filament, the crystallization rate of the composite composites increased.

Although the differences in mechanical properties were not significant, addition of NFC improved the thermostability of composites. Lignin as an antioxidant, with its UV protection capacity, improves thermal oxidative resistance and prolongs the lifetime of 3D-printed objects. The light-brown and dark-brown colors of lignin/polymerised lignin surface-modified NFC give filaments a special look and an attractive aesthetic appearance to 3D-printed objects.

## Figures and Tables

**Table 1:** Linear density and diameter of filaments; mean value ± SD.

	Linear Density (ktex) of Filaments with Different Percentages of NFC				Diameter (mm) of Filaments with Different Percentages of NFC			
	0%	1%	3%	5%	0%	1%	3%	5%
PLA	2.63 ± 0.31	-	-	-	1.62 ± 0.13	-	-	-
PLA + NFC	-	2.83 ± 0.18	2.62 ± 0.30	2.68 ± 0.29	-	1.68 ± 0.07	1.60 ± 0.09	1.68 ± 0.11
PLA + (NFC + L)	-	2.38 ± 0.06	2.33 ± 0.15	2.17 ± 0.10	-	1.52 ± 0.02	1.51 ± 0.05	1.47 ± 0.03
PLA + (NFC + PL)	-	2.05 ± 0.14	1.64 ± 0.04	1.90 ± 0.31	-	1.41 ± 0.07	1.27 ± 0.03	1.37 ± 0.11

**Table 2:** Density and sound-wave velocity; mean value ± SD.

	Density (g/cm^3^) of Filaments with Different % of NFC				Sound Wave Velocity (km/s) of Filaments with Different % of NFC			
	0%	1%	3%	5%	0%	1%	3%	5%
PLA	1.265 ± 0.072	-	-	-	2.85 ± 0.14	-	-	-
PLA + NFC	-	1.278 ± 0.047	1.300 ± 0.050	1.269 ± 0.054	-	3.29 ± 0.18	3.26 ± 0.12	2.94 ± 0.07
PLA + (NFC + L)	-	1.307 ± 0.053	1.295 ± 0.007	1.285 ± 0.045	-	3.16 ± 0.34	3.25 ± 0.26	3.17 ± 0.14
PLA + (NFC + PL)	-	1.309 ± 0.059	1.302 ± 0.034	1.277 ± 0.037	-	3.31 ± 0.10	3.42 ± 0.14	3.51 ± 0.12

**Table 3:** Tensile strength and elongation at break of filaments; mean value ± SD.

	Tensile Strength (MPa) of Filaments with Different Percentages of NFC				Elongation at Break (%) of Filaments with Different Percentages of NFC			
	0%	1%	3%	5%	0%	1%	3%	5%
PLA	56.15 ± 3.26	-	-	-	7.93 ± 2.60	-	-	-
PLA + NFC	-	50.31 ± 8.91	53.47 ± 1.28	49.94 ± 1.72	-	4.80 ± 0.44	5.51 ± 1.25	3.95 ± 0.42
PLA + (NFC + L)	-	55.14 ± 1.29	57.14 ± 5.64	52.15 ± 4.66	-	5.87 ± 1.16	4.32 ± 0.50	4.73 ± 0.75
PLA + (NFC + PL)	-	52.87 ± 1.30	51.67 ± 3.81	47.51 ± 3.17	-	5.55 ± 1.30	5.11 ± 0.77	4.19 ± 0.48

**Table 4:** Energy at break and initial modulus of filaments; mean value ± SD.

	Energy at Break (J) of Filaments with Different Percentages of NFC				Initial Modulus (GPa) of Filaments with Different Percentages of NFC			
	0%	1%	3%	5%	0%	1%	3%	5%
PLA	0.677 ± 0.259	-	-	-	1.97 ± 0.19	-	-	-
PLA + NFC	-	0.199 ± 0.042	0.420 ± 0.142	0.245 ± 0.066	-	2.22 ± 0.37	2.02 ± 0.61	1.89 ± 0.22
PLA + (NFC + L)	-	0.436 ± 0.092	0.266 ± 0.056	0.324 ± 0.114	-	2.17 ± 0.08	2.06 ± 0.14	2.08 ± 0.09
PLA + (NFC + PL)	-	0.378 ± 0.151	0.222 ± 0.049	0.149 ± 0.036	-	1.89 ± 0.21	2.09 ± 0.13	2.04 ± 0.16

**Table 5:** Compression force and bulk modulus of filaments; mean value ± SD.

	Compression Force (kN) of Filaments with Different Percentages of NFC				Bulk Modulus (MPa) of Filaments with Different Percentages of NFC			
	0%	1%	3%	5%	0%	1%	3%	5%
PLA	4.81 ± 0.35	-	-	-	102 ± 3.23	-	-	-
PLA + NFC	-	4.72 ± 0.47	4.35 ± 0.29	4.57 ± 0.23	-	97.2 ± 6.53	93.65 ± 3.79	93.24 ± 5.33
PLA + (NFC + L)	-	4.45 ± 0.09	4.59 ± 0.28	4.04 ± 0.13	-	100.9 ± 1.78	104.7 ± 5.24	94.75 ± 2.47
PLA + (NFC + PL)	-	4.01 ± 0.17	3.39 ± 0.14	3.74 ± 0.41	-	97.5 ± 1.42	92.4 ± 2.42	93.97 ± 4.29

**Table 6:** Bending force, bending stiffness, and modulus of filaments; mean value ± SD.

	Bending Force (cN) of Filaments with Different Percentages of NFC				Bending Stiffness (mN∙m) of Filaments with Different Percentages of NFC				Modulus (GPa) of Filaments with Different Percentages of NFC			
	0%	1%	3%	5%	0%	1%	3%	5%	0%	1%	3%	5%
PLA	21.37 ± 5.53	-	-	-	416 ± 134	-	-	-	1.27 ± 0.72	-	-	-
PLA+ NFC	-	21.44 ± 8.58	19.09 ± 4.75	25.27 ± 3.76	-	399 ± 155	370 ± 88.8	469 ± 64.3	-	1.04 ± 0.40	1.09 ± 0.32	1.22 ± 0.26
PLA+ (NFC + L)	-	26.23 ± 4.28	19.42 ± 5.68	18.61 ± 5.91	-	535 ± 80.3	401 ± 123	395 ± 125	-	1.81 ± 0.21	1.41 ± 0.52	1.51 ± 0.48
PLA+ (NFC + PL)	-	12.04 ± 2.90	10.71 ± 3.75	12.42 ± 2.23	-	264 ± 56.3	263 ± 92.2	283 ± 58.2	-	1.13 ± 0.25	1.56 ± 0.57	1.38 ± 0.49

**Table 7:** Bending storage modulus E’ at 40 °C and glass transition temperature Tg of filaments.

	E’ (GPa) at 40 °C of Filaments at Different Percentages of NFC				Tg * (°C) of Filaments at Different Percentages of NFC				tan δ			
	0%	1%	3%	5%	0%	1%	3%	5%	0%	1%	3%	5%
PLA	3.119	-	-	-	74.35	-	-	-	2.280			
PLA + NFC	-	3.165	3.108	3.017	-	70.84	67.48	70.84		1.814	1.704	2.083
PLA + (NFC + L)	-	3.332	3.161	3.181	-	68.26	67.61	68.84		2.536	0.382	2.107
PLA + (NFC + PL)	-	3.847	3.241	4.276	-	69.26	72.15	70.45		2.065	2.546	2.180

* Tg was indicated as a peak of tan δ.

**Table 8:** Temperature of cold crystallization and melting by DSC analysis.

	Tcc (°C) of Filaments at Different Percentages of NFC				Tm (°C) of Filaments at Different Percentages of NFC			
	0%	1%	3%	5%	0%	1%	3%	5%
PLA	126.1				149.3			
PLA + NFC		116.4	116.8	117.7		147.6	147.1	148.1
PLA + (NFC + L)		117.9	116.4	118.4		148.2	147.3	147.7
PLA + (NFC + PL)		120.0	120.0	119.7		147.9	147.8	147.6

**Table 9:** Enthalpy of cold crystallization, enthalpy of melting, and degree of crystallinity by DSC analysis.

	ΔHcc (J/g) of Filaments at Different Percentages of NFC				ΔHm (J/g) of Filaments at Different Percentages of NFC				Xcc (%) of Filaments at Different Percentages of NFC			
	0%	1%	3%	5%	0%	1%	3%	5%	0%	1%	3%	5%
PLA	5.94				5.4				6.3			
PLA + NFC		21.4	21.0	18.9		19.7	20.7	19.6		23.1	23.1	21.4
PLA + (NFC + L)		20.0	22.2	19.9		21.2	21.4	20.0		21.6	24.5	22.4
PLA + (NFC + PL)		19.3	19.1	19.5		18.8	18.0	18.8		20.8	20.9	21.9

## Data Availability

The data presented in this study are available on request from the corresponding author.
